# Transcriptional analysis of the jamaicamide gene cluster from the marine cyanobacterium *Lyngbya majuscula *and identification of possible regulatory proteins

**DOI:** 10.1186/1471-2180-9-247

**Published:** 2009-12-01

**Authors:** Adam C Jones, Lena Gerwick, David Gonzalez, Pieter C Dorrestein, William H Gerwick

**Affiliations:** 1 Center for Marine Biotechnology and Biomedicine, Scripps Institution of Oceanography, University of California San Diego, 9500 Gilman Drive, La Jolla, CA 92093 USA; 2 Department of Chemistry, University of California San Diego, 9500 Gilman Drive, La Jolla, CA 92093 USA; 3 Department of Biochemistry, University of California San Diego, 9500 Gilman Drive, La Jolla, CA 92093 USA; 4 Department of Pharmacology, University of California San Diego, 9500 Gilman Drive, La Jolla, CA 92093 USA; 5 Skaggs School of Pharmacy and Pharmaceutical Sciences, University of California San Diego, 9500 Gilman Drive, La Jolla, CA 92093 USA

## Abstract

**Background:**

The marine cyanobacterium *Lyngbya majuscula *is a prolific producer of bioactive secondary metabolites. Although biosynthetic gene clusters encoding several of these compounds have been identified, little is known about how these clusters of genes are transcribed or regulated, and techniques targeting genetic manipulation in *Lyngbya *strains have not yet been developed. We conducted transcriptional analyses of the jamaicamide gene cluster from a Jamaican strain of *Lyngbya majuscula*, and isolated proteins that could be involved in jamaicamide regulation.

**Results:**

An unusually long untranslated leader region of approximately 840 bp is located between the jamaicamide transcription start site (TSS) and gene cluster start codon. All of the intergenic regions between the pathway ORFs were transcribed into RNA in RT-PCR experiments; however, a promoter prediction program indicated the possible presence of promoters in multiple intergenic regions. Because the functionality of these promoters could not be verified *in vivo*, we used a reporter gene assay in *E. coli *to show that several of these intergenic regions, as well as the primary promoter preceding the TSS, are capable of driving β-galactosidase production. A protein pulldown assay was also used to isolate proteins that may regulate the jamaicamide pathway. Pulldown experiments using the intergenic region upstream of *jamA *as a DNA probe isolated two proteins that were identified by LC-MS/MS. By BLAST analysis, one of these had close sequence identity to a regulatory protein in another cyanobacterial species. Protein comparisons suggest a possible correlation between secondary metabolism regulation and light dependent complementary chromatic adaptation. Electromobility shift assays were used to evaluate binding of the recombinant proteins to the jamaicamide promoter region.

**Conclusion:**

Insights into natural product regulation in cyanobacteria are of significant value to drug discovery and biotechnology. To our knowledge, this is the first attempt to characterize the transcription and regulation of secondary metabolism in a marine cyanobacterium. If jamaicamide is light regulated, this mechanism would be similar to other cyanobacterial natural product gene clusters such as microcystin LR. These findings could aid in understanding and potentially assisting the management of toxin production by *Lyngbya *in the environment.

## Background

Over the past 30 years, the search for bioactive secondary metabolites (natural products) from marine organisms has yielded a wealth of new molecules (estimated at ~17,000) with many fundamentally new chemotypes and extraordinary potential for biomedical research and applications [[1], and previous references therein]. Marine cyanobacteria continue to be among the most fruitful sources of marine natural products, with nearly 700 compounds described [2,3]. The filamentous marine cyanobacterium *Lyngbya majuscula *(Gomont) is of particular importance, as approximately 35% of all cyanobacterial bioactive compounds have been reported from the genus *Lyngbya*, with 76% of these coming from *L. majuscula *[3]. More recently, compound isolation and structure elucidation from *L. majuscula *has been complemented with the characterization of biosynthetic gene clusters that encode a number of these compounds. The gene clusters for several potent anticancer and neurotoxic agents such as curacin A, barbamide, and the jamaicamides have provided new insight into the biosynthetic strategies and logic used by this organism for compound production, as well as unique enzymes involved in unprecedented molecular tailoring reactions [4-7].

Despite considerable interest in pursuing cyanobacterial lead compounds as potential drug candidates, an adequate supply of these compounds for clinical research is often impossible to obtain without impractically large scale field collections or sophisticated and expensive synthetic methods [8,9]. With some notable examples [10-13] it has been difficult to induce microbial gene clusters to produce their natural products in heterologous hosts, and thus this technology is not currently predictable [14]. Equally problematic, filamentous marine cyanobacteria such as *Lyngbya *grow slowly in laboratory culture, with doubling times in some cases of about 18 days [15].

One avenue for increasing compound production from marine cyanobacteria could be to take advantage of regulatory elements associated with a biosynthetic gene cluster of interest. Although genetic controls of several primary metabolic functions in cyanobacteria including circadian rhythms [16], heterocyst development [17], and nutrient uptake [18] have been described, information regarding transcriptional regulation of cyanobacterial secondary metabolites is currently limited to freshwater toxins such as the microcystins. The microcystins are potent hepatotoxins synthesized by several freshwater cyanobacteria of worldwide occurrence [19] and are generated via a mixed polyketide synthase/non-ribosomal peptide synthetase (PKS/NRPS) gene cluster [20]. Expression of the microcystin gene cluster is positively correlated with increased light intensity and red light in particular [21]. Moreover, the gene cluster has different transcription start sites depending on light levels [22]. Other environmental factors have been evaluated for their effects on microcystin production, and increasing evidence suggests that iron may be important. Transcription of genes from the microcystin gene cluster increases with iron starvation [23], and in the presence of iron, a ferric uptake regulator (Fur) protein appears to bind to the microcystin bidirectional promoter and may decrease microcystin production [24]. Because it complexes with iron and other metals [25] microcystin may therefore function as a siderophore. Alternatively, microcystin has been proposed to serve in intraspecies communication, where release of the compound is interpreted as cell death by other *Microcystis *sp. and causes increased microcystin production to enhance localized toxicity [26].

As with microcystin, many of the toxins found in *L. majuscula *are also produced by gene clusters comprised of PKS/NRPS architecture. PKS/NRPS gene clusters in other bacteria have been found to include imbedded regulatory proteins, such as the ***S****treptomyces ***A**ntibiotic **R**egulatory **P**roteins (SARPs) found within the confines of several antibiotic pathways in *Streptomyces *[27]. However, cyanobacterial natural product gene clusters identified to date do not contain any apparent associated regulatory proteins.

Insight into the mechanisms used by *L. majuscula *in the transcription of secondary metabolite gene clusters could be of significant value in enhancing the overproduction of potential drug leads in laboratory culture. Increased compound yield would reduce the need and environmental impact of repeated large scale field collections or the time and expense of chemical synthesis. Additionally, because the secondary metabolite biosynthetic gene clusters identified thus far from *L. majuscula *have been from different strains of the same species, transcription of each pathway could be under similar mechanisms of regulation.

This paper provides an analysis of transcriptional regulatory elements associated with the jamaicamide gene cluster from *Lyngbya majuscula*, and to our knowledge is the first such effort for a secondary metabolite gene cluster from a marine cyanobacterium. The jamaicamides are mixed PKS/NRPS neurotoxins that exhibit sodium channel blocking activity and fish toxicity. The molecules contain unusual structural features including a vinyl chloride and alkynyl bromide [6]. The gene cluster encoding jamaicamide biosynthesis is 57 kbp in length, and is composed of 17 ORFs that encode for proteins ranging in length from 80 to 3936 amino acids. Intergenic regions between 5 and 442 bp are located between all but two of the ORFs, and a region of approximately 1700 bp exists between the first jamaicamide ORF (*jamA*, a hexanoyl ACP synthetase) and the closest upstream (5') ORF outside of the cluster (a putative transposase). In this study, we used RT-PCR to locate the transcriptional start site (TSS) of the jamaicamide gene cluster. Because it is not yet possible to perform genetics in filamentous marine cyanobacteria such as *Lyngbya*, we used a reporter gene assay to identify several possible internal pathway promoters. We also isolated at least one possible regulatory protein using pulldown experiments that is able to bind to the region upstream of the transcription start site in gel shift assays. Bioinformatic analyses conducted with the protein sequence suggest a correlation between secondary metabolite production and complementary chromatic adaptation (CCA) in cyanobacteria.

## Results

### RT-PCR using *L. majuscula* RNA to search for the transcriptional start site (TSS) and promoter regions in the jamaicamide pathway

**Figure 1:**
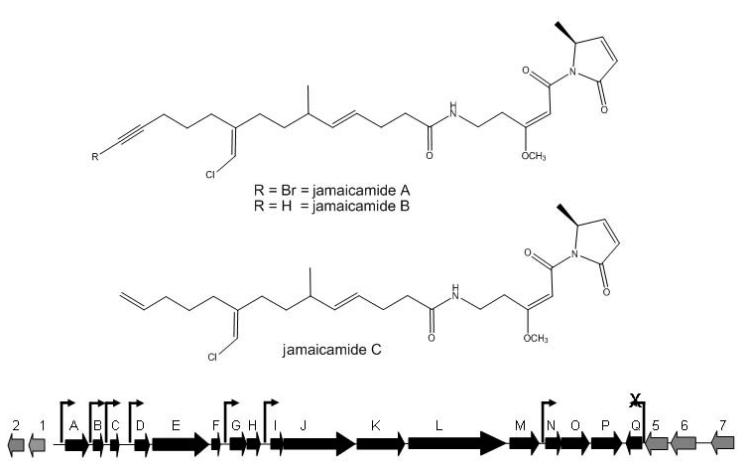
**Structures of the jamaicamides and the jamaicamide biosynthetic gene cluster **[6]. Genes associated with the pathway are represented by black arrows, and genes flanking the pathway are represented in gray. Elevated arrows above the upstream regions of selected open reading frames indicate where promoter activity was detected using the β-galactosidase reporter assay. The region upstream of *jamQ *did not have any detectable promoter activity in the assay.

**Figure 2:**
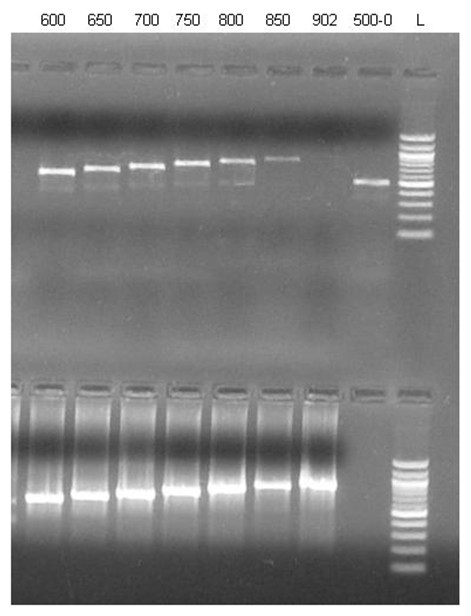
**Transcription start site (TSS) primer extension experiment using first strand cDNA upstream of *jamA *(top) or jam fosmid (bottom) as PCR templates**. The upstream region sizes (e.g., 600-0, 650-0) are indicated above each lane.

**Figure 3:**
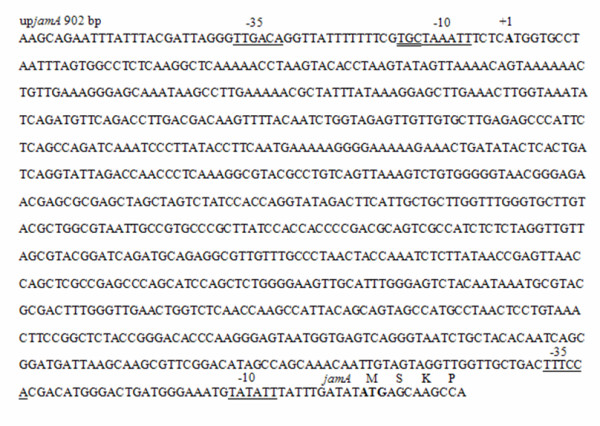
**Location of identified promoter regions and transcription start site (TSS) upstream of *jamA***. The consensus -35 and -10 boxes of each region are underlined. The conserved extended -10 TGn box of the primary pathway promoter is double underlined. The putative TSS is noted at +1, and was chosen based on similarities to the consensus *E. coli *TSS nucleotide region [29]. The first four codons of the *jamA *gene are noted at the end of the sequence.

The initial characterization of the jamaicamide gene cluster [6] described that the first 16 ORFs of the gene cluster (*jamA-jamP*) are all transcribed in the forward direction, while the last ORF (*jamQ*, a putative condensation domain thought to be involved in the cyclization of the pyrrolinone ring of the molecule) is transcribed in the reverse direction (Figure 1). In order to determine the location of the transcriptional start site (TSS) of the gene cluster, RNA was isolated from the jamaicamide producing strain of *Lyngbya majuscula *(JHB). First strand cDNA was synthesized using reverse transcriptase and a reverse primer designed as a complement to the 5' end of the *jamA *gene (Additional file 1: Table S1). Initial experiments creating second strand cDNA using the first strand cDNA as template found that an unusually long untranslated leader region of at least 500 bp preceded *jamA*. A primer extension experiment was conducted in which second strand cDNA was amplified in 50 bp increments beyond this 500 bp location. The experiment indicated that transcription of RNA began between 850 bp and 902 bp upstream of the *jamA *ORF start site (Figure 2). Using comparisons to consensus promoter and transcription start regions in *E. coli *[28-30], a putative promoter was identified which, relative to a probable TSS (844 bp upstream of *jamA*), included conserved hexamer RNA polymerase (RNAP) binding sites at -35 and -10 bp, a conserved extended -10 TGn region upstream of the -10 box, and an optimal DNA length between the hexamers (17 bp) (Figure 3).

We also evaluated whether the jamaicamide gene cluster contained non-transcribed intergenic regions between ORFs that could indicate the presence of breaks in the transcripts. Primers designed for those intergenic regions in the pathway 20 bp in size or larger (all but 2 intergenic regions) were used for synthesizing and amplifying cDNA to determine whether they were transcribed. All of the intergenic regions in the jamaicamide pathway tested were amplified into second strand cDNA, including the intergenic region between *jamP *and *jamQ*. Intergenic regions between the two ORFs downstream of *jamQ *(putative transposases) were also transcribed. These results indicated that the majority of the jamaicamide gene cluster is composed of the operon *jamABCDEFGHIJKLMNOP*. Because no apparent breaks in transcription occurred between *jamQ *and at least the two neighboring downstream transposases (*ORF5 *and *ORF6*) and a hypothetical protein (*ORF7*), one contiguous transcript may encode the translation of all of these proteins. Transcription of the intergenic region between *jamP *and *jamQ *indicated that a transcript including *jamP *must extend at least into the complementary strand of *jamQ *before termination, although transcription in the opposite direction would be necessary to generate *jamQ *mRNA.

### Use of promoter prediction and β-galactosidase reporter gene assays to search for promoter activity

The large size (approximately 55 kbp) of the main jamaicamide operon (*jamA-P*) suggested that multiple promoters would likely be needed for efficient jamaicamide transcription. Because transcripts were found for each of the intergenic regions between the ORFs, these promoters may function intermittently and could be subject to promoter occlusion [22]. A software prediction program (BPROM, http://www.softberry.com) was used to predict whether the intergenic regions from the jamaicamide pathway contained conserved promoter binding regions. Several of these regions were predicted to contain at least one potential pair of -35 and -10 binding sites (Table 1). Because transformation methods into *L. majuscula *have not yet been developed, we used a reporter gene assay in *E. coli *to determine whether any of these upstream (up-) regions could function as promoters. Each region predicted to contain a promoter (up*jamA*, up*jamB*, up*jamC*, up*jamD*, up*jamG*, up*jamI*, up*jamN*, and up*jamQ*), as well as the promoter upstream of the jamaicamide TSS, was amplified with specific primers from fosmids containing different portions of the jamaicamide biosynthetic pathway ([6]; Additional file 1: Table S1). Each of these regions were individually ligated into the pBLUE TOPO vector (Invitrogen) and transformed into TOP-10 *E. coli*. The resulting constructs were evaluated for relative promoter activity using the β-galactosidase reporter gene assay (Invitrogen), standardized against total soluble protein content measured by BCA assay (Pierce). For up*jamA*, two regions were evaluated, including the region predicted to contain the initial promoter, as well as immediately upstream of the *jamA *gene (a region with high activity in preliminary assays). The arabinose promoter from *E. coli *was amplified from the pBAD vector (Invitrogen) and ligated into the pBLUE vector as a positive control, while a 49 bp segment of a jamaicamide pathway gene (*jamG*) ligated into pBLUE vector was used as a negative control.

**Table 1:** Predicted -35 and -10 promoter regions (bold) and transcription start sites (TSS; nucleotides in bold italics at the end of each sequence) for intergenic regions in the jamaicamide gene cluster (accession #AY522504).

Upstream region of gene	Predicted TSS location (bp)	ORF start (bp)			
**up*jamA***	6626	6630	CTGAC**TTTCCA**CGACATGGGACTGATGGGAAATG**TATATT**TATTTG***A***		
**up*jamB***	8464	8591	GTGGG**TTGATT**TGATCAAGTTTGATGA**TATAAT**TTGATTT***A***		
**up*jamB***	8501	8591	TTTAA**TTTACA**GGGATACCGCCAATTCGG**TAACCT**GGAAA***A***		
**up*jamC***	9614	9718	AAAAC**TTGTCA**ACCTGAACAAGATCCTGAA**CAAAAT**ATTGTT***G***		
**up*jamD***	10433	10463	ACAGT**TTGATG**GTGCCGCTATTTTGAAGTTG**GAAAAT**TTTTT***A***		
**up*jamG***	18145	18222	ATTTG**TTGTTT**GGGAATCGGGAATTGG**TATTAG**TAGTGGA***A***		
up*jamI*	20776	20982	CGGAA**TTCAAA**ATTCAAAATTCAAAATGCTTATG**GATTAT**GGAGTAA***A***		
**up*jamI***	20989	20982	CCAGG**TTGACA**AACCATTGATAAAGC**TATAGT***ATG*TATT***A***		
**up*jamN***	51787	51811	TGGAG**TATAAA**AAACAGAGCCTGGT**GATAGT**TAATTA***A***		
up*jamQ*	63710^a^	63646^a^	GAACT**TTGAAT**CCTCTATTTTGAT**TAAATT**TGGAG***A***		

^a^: Numbers correspond to bp in complementary 3' - 5' direction.

Bold -35 and -10 binding regions were predicted using BPROM (softberry.com) in comparison to the *E. coli* σ^70^ consensus -35 (TTGACA) and -10 (TATAAT) promoter regions.  If upstream regions had more than one predicted promoter region, the region receiving the highest predicted score is provided (with the exception of up*jamB*, which had two high scoring regions, and up*jamI*, in which both predicted promoters were tested in the β-galactosidase assay). Each upstream region listed in the first column had activity in the reporter assay, except those not shown in bold text.  The italic ATG in the second up*jamI* predicted promoter region indicates the *jamI* start codon.  TSS nucleotides were predicted to be A or G based on comparisons to the most common TSS nucleotides in *E. coli*[29].

**Figure 4:**
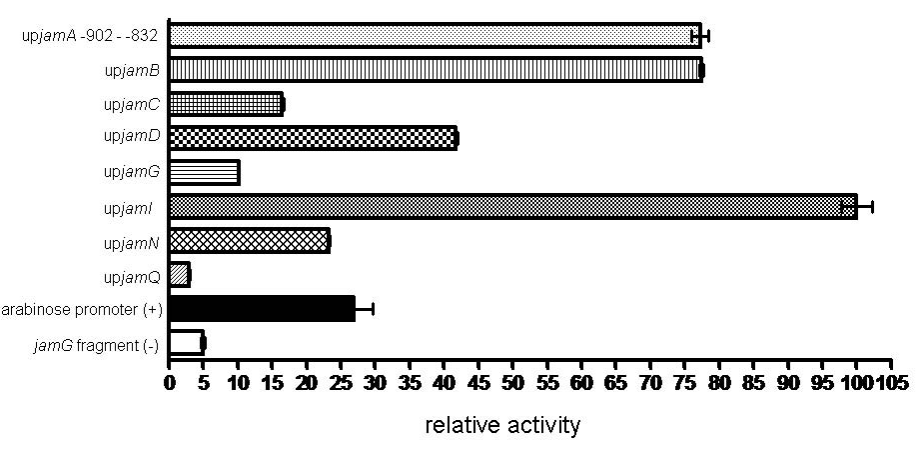
**Relative activity of the primary promoter upstream of *jamA *and predicted promoters in jamaicamide intergenic regions in the β-galactosidase reporter assay**. Standard error is represented by error bars.

Several of the tested intergenic regions exhibited significantly stronger promoter activity than the positive control, including the promoter identified from the primer extension experiment (up*jamA*-902 - -832 bp), as well as up*jamB*, up*jamD*, and up*jamI *(Figure 4). The intergenic regions up*jamG *and up*jamN *both had some promoter activity, although lower than the positive control. The region upstream of *jamQ *did not have any detectable promoter activity in the assay, which suggested that the promoter for this transcript may be located upstream of an adjacent ORF.

**Figure 5:**
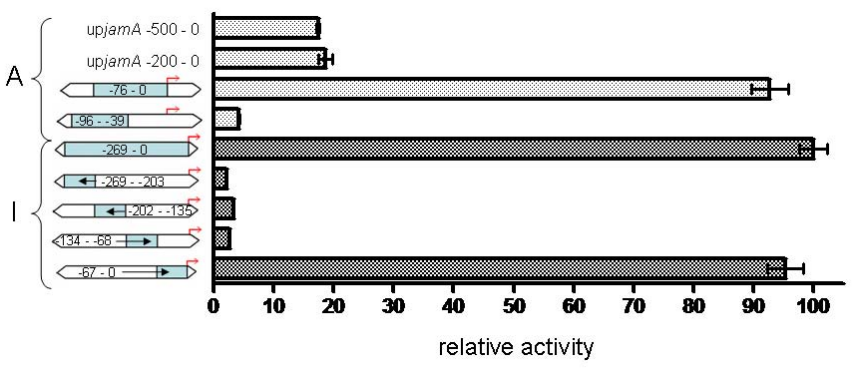
**Activity of truncated up*jamA *and up*jamI *regions in the β-galactosidase assay**. Trimmed regions are represented by blue shaded figures with associated base pair numbers. Red arrows indicate the start codon of the downstream ORF (*jamA *or *jamI*). Relative activity was calculated on same scale as Figure 4. Standard error is represented by error bars.

To more precisely localize the promoter regions upstream of two of these genes, a series of additional assays were conducted using truncated regions of upjam*A *(immediately upstream of the *jamA *gene) and up*jamI*. For up*jamA*, promoter activity was comparable relative to the positive control when testing longer upstream fragments (-500 - 0 and -200 - 0 bp; Figure 5). However, when small fragments closer to the *jamA *ORF start site were used, the promoter activity increased significantly, with maximal activity observed for the fragment -76 - 0 bp upstream of *jamA*. The promoter in the -76 - 0 region appeared to require the sequence fragment -38 - 0, as another construct containing the region up*jamA*-96 - -38 did not have any promoter activity. The entire 269 bp up*jamI *upstream region also displayed strong promoter activity relative to the positive control. Promoter activity was lost using fragments encompassing -269 - -68 bp, but restored using the fragment -67 - 0 bp (Figure 5). Inspection of the sequences included in these active, truncated regions of up*jamA *and up*jamI *led to the identification of possible conserved promoter elements in close proximity to the ORF start sites for both genes (Table 1).

**Figure 6:**
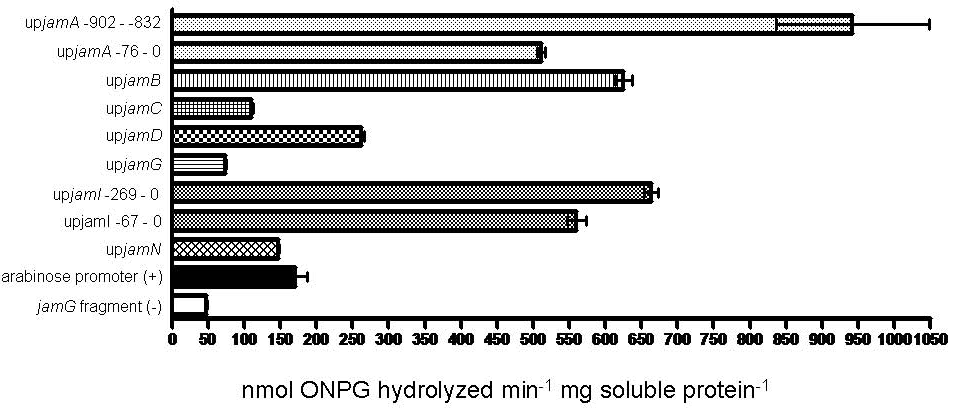
**Specific activity of the strongest promoters in the β-galactosidase assay**. Base pair number relative to gene ORF start site is provided when necessary. Standard error is represented by error bars.

To quantitatively determine the promoter activities of the DNA fragments, a series of β-galactosidase assays incorporating a serial dilution of *E. coli *soluble protein lysate was also used in order to avoid saturation problems in color development (Figure 6). These data were used to calculate β-galactosidase activity in terms of nmol ONPG hydrolyzed min^-1 ^mg soluble protein^-1 ^for each of the upstream fragments with any detectable promoter activity. The strongest promoter was the section upstream of the jamaicamide TSS (-902 - -832 upstream of *jamA*), with an average of approximately 950 nmol ONPG hydrolyzed min^-1 ^mg soluble protein^-1^. The promoter immediately upstream of *jamA *(-76 - 0) and those upstream of *jamB, jamD*, and *jamI *yielded lower values, with up*jamA*, up*jamB *and up*jamI *between 500-700 nmol ONPG hydrolyzed min^-1 ^mg soluble protein^-1^, and up*jamD *at approximately 265 nmol ONPG hydrolyzed min^-1 ^mg soluble protein^-1^. Reduced activity was found for promoters upstream of *jamC*, *jamG*, and *jamN*, with values ranging from approximately 75 to 150 nmol ONPG hydrolyzed min^-1 ^mg soluble protein^-1^. The arabinose promoter positive control construct yielded an average value of 170 nmol ONPG hydrolyzed min^-1 ^mg soluble protein^-1^.

### Isolation and characterization of possible transcription factors from a pulldown assay

**Figure 7:**
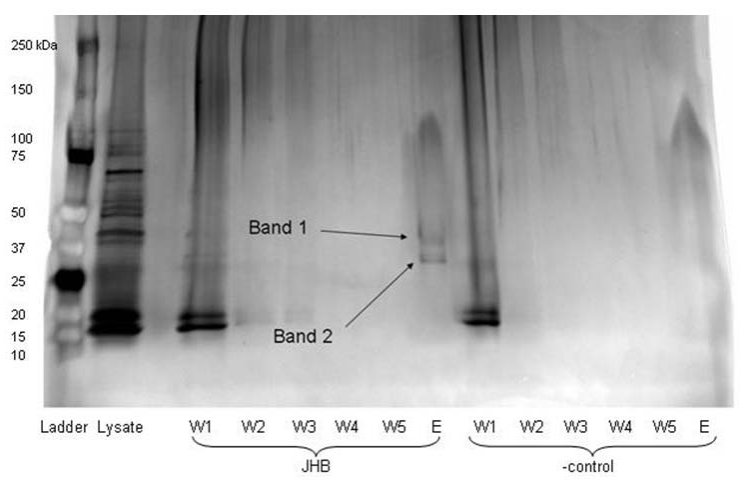
**Results from JHB soluble protein pulldown experiment**. From left to right: Ladder, JHB soluble protein lysate, wash fractions (W1 - 5) and elution (E) for incubations with the 1020 bp probe (labeled JHB) or without probe (labeled -control). Note the presence of two bands eluted from the beads containing the probe, indicating successful binding of possible regulatory proteins to the upstream region of *jamA*.

To determine whether jamaicamide regulatory proteins are encoded in the *L. majuscula *JHB genome, we performed DNA - protein "pulldown" experiments to isolate proteins with affinity to the upstream region of *jamA*. A biotinylated, DNA probe extending from 1000 bp upstream of *jamA *to 20 bp into the *jamA *gene (encompassing both the putative promoter region and entire untranslated leader region; primers in Additional file 1: Table S1) was used to label streptavidin coated magnetic Dynabeads (Invitrogen), which were then incubated with a soluble protein lysate from *L. majuscula *JHB. A series of wash steps were first conducted to remove proteins non-specifically bound, followed by elution of those proteins specifically bound to the probe. This elution was visualized using SDS-PAGE and revealed at least two bands of approximately 30-45 kDa in size (Figure 7). The protein bands from the gel, as well as crude fractions eluted from the magnetic beads in repeated experiments, were analyzed with LC-MS/MS.

The fragmented peptides generated from the LC-MS/MS analysis of the gel bands were used to query the unfinished *Lyngbya majuscula *3L genome (a strain from Curaçao that produces several natural products, including barbamide and curacin A) using the MS/MS post-processing program InSpecT [31]. By this approach, two proteins were identified with high confidence from "band 2" (Figure 7), which had a global distribution (N-terminal to C-terminal) among the identified peptides: (i) All4300 protein (39.2% coverage and a molecular weight of 32 kDa), and (ii) hypothetical protein (35.9% coverage and a molecular weight of 33 kDa). Manual annotation of the most abundant peptide identified within the primary sequence of All4300 demonstrated the b and y ion series fell within a mass error of 5-400 ppm. Furthermore, the b and y-ion series for this peptide showed 22/30 possible fragmentations covered with several contingent ion series. The ion series for the hypothetical protein showed similar results to the All4300 protein. Results from the LC-MS/MS of the PAGE gel "band 1" (Figure 7) were inconclusive. Separate analyses of the elution fractions identified with high confidence the same All4300 and hypothetical protein from band 2, as well as a number of putative proteins in the 3L genome such as a peptidase (~45 kDa) and an AP endonuclease (~30 kDa). Several pigment related proteins were also identified that were not visually apparent by SDS-PAGE (smaller than the two main bands indicated on Figure 7), including C-phycoerythrin class 1 subunit alpha (~19 kDa), allophycocyanin alpha subunit (~17 kDa), and photosystem I (PsaD) (~16 kDa).

BLAST analyses of the All4300 and the hypothetical protein (referred to subsequently as protein 5335 and protein 7968, respectively, from the *L. majuscula *3L genome; annotations in progress) both yielded a number of hypothetical protein matches in other cyanobacteria including *Anabaena variabilis, Microcoleus chthonoplastes*, *Nostoc punctiforme*, and *Trichodesmium erythraeum *(JHB protein BLAST hits in Table 2; see below). Interestingly, both proteins also matched (although significantly better for 7968) with the protein RcaD, an activator protein from the cyanobacterium *Calothrix *(= *Fremyella diplosiphon *or *Tolypothrix*) known to regulate complementary chromatic adaptation [32-35]. Complementary chromatic adaptation (CCA) is a phenomenon exhibited by many cyanobacteria in response to changes in light wavelength and intensity. CCA allows cyanobacteria to alter pigment levels so as to optimize their capacity for photosynthesis, and usually involves variation between green and red phenotypes [36]. RcaD is a protein that binds to the promoter for phycocyanin 2 (*cpc2*) and alters the expression of several red light operons in the acclimation phase of CCA [34,35]. Another protein, RcaG, is located downstream of RcaD and has been identified as a putative ATPase. RcaG may facilitate binding of RcaD to DNA, and could require phosphorylation to complete this task [34]. Bioinformatic analysis of the *L. majuscula *3L genome revealed that the proteins immediately downstream of 5335 and 7968 both resulted in BLAST hits with RcaG, although as with RcaD, the protein neighboring 7968 (7969) had much stronger sequence identity than the neighboring protein to 5335 (5336).

**Table 2:** BLAST results with *Lyngbya majuscula *JHB proteins 5335 and 7968.

5335 (279 aa)						
**Best BLAST hit**	**BLAST organism**	**Size (aa)**	**identity**	**similarity**	**e value**	**accession #**
hypothetical protein	*Nostoc punctiforme *PCC 73102	217	56	70	8.00E-62	YP_001867255
hypothetical protein	*Microcoleus chthonoplastes *PCC 7420	245	56	71	2.00E-59	ZP_05025825
hypothetical protein all4300	*Nostoc *sp. PCC 7120	227	49	68	4.00E-54	NP_488340
hypothetical protein	*Anabaena variabilis *ATCC 29413	221	49	65	1.00E-51	YP_321771
hypothetical protein	*Lyngbya *sp. PCC 8106	224	47	64	9.00E-47	ZP_01623947
hypothetical protein	*Lyngbya *sp. PCC 8106	156	33	56	2.00E-11	ZP_01621638
hypothetical protein	*Nodularia spumigena *CCY9414	100	41	61	3.00E-11	ZP_01628571
hypothetical protein	*Arthrospira maxima *CS-328	131	32	60	1.00E-08	ZP_03271683
**RcaD protein**	***Tolypothrix *sp. PCC 7601**	**285**	**22**	**48**	**0.2**	CAC39267
						
7968 (304 aa)						
**Best BLAST hit**	**BLAST organism**	**Size (aa)**	**identity**	**similarity**	**e value**	**accession #**
hypothetical protein	*Cyanothece *sp. PCC 7424	274	49	69	2.00E-68	YP_002380360
**RcaD protein**	***Tolypothrix *sp. PCC 7601**	**285**	**43**	**63**	**3.00E-54**	CAC39267
hypothetical protein	*Trichodesmium erythraeum *IMS101	272	40	59	3.00E-52	YP_720119
hypothetical protein	*Nodularia spumigena *CCY9414	280	44	62	1.00E-50	ZP_01631082
hypothetical protein	*Microcoleus chthonoplastes *PCC 7420	287	41	62	7.00E-50	ZP_05025219
hypothetical protein	*Synechococcus *sp. PCC 7335	199	33	57	3.00E-22	ZP_05035072

Primers were designed from each of the gene sequences for the two proteins identified above using the *L. majuscula *3L unfinished genome, and were successful in amplifying homologous gene sequences from *L. majuscula *JHB genomic DNA. The JHB homolog to 5335 encodes for a protein that differs from the 3L protein by only one amino acid (99.6% identical), while the 7968 homolog in JHB encodes for a protein 89.5% identical to the 7968 protein in 3L. Alignments of each JHB protein with their nearest respective BLAST hits (alignment of protein 7968 shown in Additional file 2: Figure S1) indicated several conserved sequence regions, with the highest level of conservation found toward the C terminal end of the proteins (a region in the RcaD protein thought to be involved in DNA binding) [34].

### Recombinant expression of identified proteins and Electromobility Shift Assays (EMSAs)

**Figure 8:**
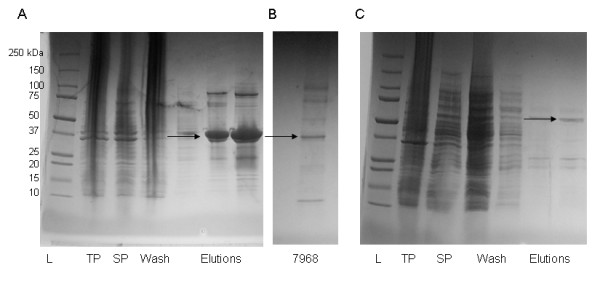
**Recombinant expression of JHB proteins**. A: Protein expression from *L. majuscula *JHB 7968 (His+protein: ~37 kDa). Arrow indicates eluted protein. B: Protein 7968 after thrombin His tag cleavage and concentration. Arrow indicates cleaved protein. C: Protein expression from *L. majuscula *JHB 5335 GST fusion vector (GST+protein: ~60 kDa). Arrow indicates eluted GST+5335 protein.

**Figure 9:**
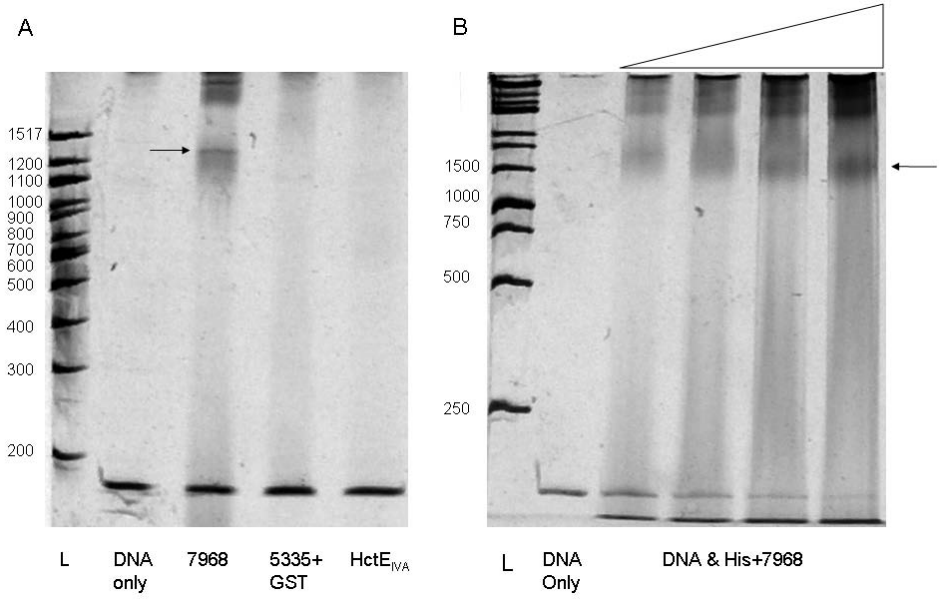
**Electromobility shift assays**. A) EMSA gel shift assay with DNA region -1000 - -832 bp upstream of *jamA*. DNA [270 fmol (= 30 ng)] was assayed with (from left to right) no protein, 7.3 pmol of 7968, 8.4 pmol of GST+5335, or 31 pmol of HctE_IVA_. Arrow indicates DNA + protein shift for 7968. B) Serial titration experiment with 45 fmol (= 5 ng) of the same DNA region with (from left to right) no protein, 6.8 pmol, 13.7 pmol, 27.3 pmol, or 54.8 pmol His+7968. Arrow indicates DNA + protein shift.

The sequences encoding the 5335 and 7968 proteins in JHB were used in creating constructs for recombinant expression in *E. coli *(Figure 8). After expression and purification of each protein, both were used in **E**lectro**m**obility **S**hift **A**ssays (EMSAs). In these assays, protein and a fragment of DNA amplified from a region that included both the sequence of the primary jamaicamide promoter and the region upstream from the original probe (1000 - 832 bp upstream of *jamA*) were incubated and visualized on native PAGE gels. Recombinant 7968 was found to bind this putative transcription factor binding region upstream of *jamA *after His tag removal with thrombin cleavage (Figure 9a), although promiscuous binding was also observed with other control DNA fragments (data not shown). A serial titration of 7968 with the N-terminal His tag still attached showed increased DNA binding with larger amounts of protein (Figure 9b). Recombinant protein 5335 was expressed and purified with a GST-tag on the N-terminus of the protein. However, attempts to remove the GST tag were unsuccessful, and thus we assayed protein 5335 with the GST tag still attached (Figure 8c). This version of 5335 did not bind to the *upjamA*-1000 - -832 bp region (Figure 9a), even with elevated protein concentrations (Additional file 3: Figure S2).

## Discussion

In this study, we explored the transcriptional machinery associated with the jamaicamide biosynthetic gene cluster in *Lyngbya majuscula*. The jamaicamide cluster was chosen because it possesses a number of features commonly seen in other secondary metabolites isolated from marine cyanobacteria [3]. The jamaicamides are produced by the most prolific cyanobacterial natural product producer yet known (*L. majuscula*), are bioactive (ichthyotoxic, neurotoxic), are composed of mixed PKS/NRPS derived subunits, and contain unusual structural features such as a vinyl chloride and alkynyl bromide rarely seen in natural products from other organisms.

The first description of the jamaicamides [6] demonstrated that the cluster is composed of 17 ORFs, with 16 transcribed in the same direction. The cluster is flanked on the 5' and the 3' ends by transposases and hypothetical proteins. From the results of our RT-PCR experiments, it appears that the gene cluster is preceded by an unusually long untranslated leader region (at least 844 bp), one that may be unprecedented in size for a secondary metabolite gene cluster. The function of having such a long region between the TSS and the start codon of *jamA *is unclear at this time, but may be important for overall regulation of the pathway. In *Synechococcus *PCC 7942, the psBAII and psBAIII genes encoding the photosystem II reaction center D1 protein have *cis *regulatory elements in addition to basal promoters. Contained in the untranslated leader region downstream of the psB TSS are light responsive elements that were found to be responsible for increased expression of the genes under high light conditions [37]. In the jamaicamide pathway, the fact that another region of DNA immediately upstream of *jamA *can function as a strong promoter indicates that although transcription may initiate well before the ORF start site, there could be a supplemental means of boosting transcription closer to the first protein in the cluster.

The amplification of second strand cDNA from JHB RNA corresponding to all of the intergenic regions between the jamaicamide ORFs tested indicated that the pathway is transcribed in at least two pieces. The first, *jamABCDEFGHIJKLMNOP*, is sufficiently large (~55 kb) to assume that multiple transcripts could be needed to process this portion of the gene cluster. A similar situation was found with the microcystin gene cluster [22], in which all of the intergenic regions of the pathway aside from the bidirectional promoter were transcribed, and RACE experiments with several of these regions detected variations in intergenic TSS locations. As with microcystin, the jamaicamide pathway could contain internal promoters which, while not representing true breaks in the transcription of the pathway, can function independently if not overwritten by RNAP acting from an upstream promoter (promoter occlusion; [38]). Indeed, several of these regions were able to function as promoters in a reporter assay (see below).

A second transcript in the direction complementary to the large transcript in the jamaicamide pathway is probably needed to include *jamQ*, a gene encoding a condensation like protein that is likely involved with the creation of the pyrrolinone ring of the molecule. According to our RT-PCR experiments, the regions between *jamQ *and the three genes closest upstream (*ORF5 *and *ORF6*, both transposases, and *ORF7*, a hypothetical protein), are all transcribed. In addition, the upstream region of *jamQ *does not appear to serve as a strong promoter in β-galactosidase reporter assays (see below), despite the presence of possible conserved promoter domains (Table 1). From these data, it appears that *jamQ *could be part of a larger transcript including these transposases. A larger intergenic region (approximately 1070 bp) lies upstream of *ORF7*, which could contain the TSS and a promoter for this transcript. The reason for including at least one transposase in the *jamQ *transcript is unclear, but this may be a way of ensuring transposable elements have remained associated with the cluster so as to facilitate horizontal gene transfer and pathway evolution. The hectochlorin biosynthetic gene cluster from *L. majuscula *JHB [39] contains a transposase (*hctC*) located between two of the initial genes (*hctB and hctD*) in the pathway, which is also thought to contribute to the plasticity of the cluster.

Biosynthetic investigations using *Lyngbya majuscula *strains have been highly successful in identifying secondary metabolite gene clusters, in part because *L. majuscula *readily incorporates isotopically labeled precursors in feeding studies [5,6]. However, further experimentation by way of gene knockout or overexpression in *L. majuscula *is not yet possible because a viable means of genetic transformation has not been developed. Due to this limitation, we used genetic constructs in *E. coli *to determine whether the promoters identified in this study, including the primary pathway promoter upstream of the TSS and those predicted in intergenic regions, were functional. Although some differences exist in the structure of RNAP between the two bacteria [40], promoter structures in cyanobacteria are often compared to consensus sequences in *E. coli *[22,41]. Furthermore, a strong *E. coli *promoter has been shown to function in the cyanobacterium *Synechococcus *[37] and the psb2 promoter from *Microcystis *can be used in *E. coli *to drive β-galactosidase production [42]. The reporter assay proved effective in verifying the promoter identified upstream of the jamaicamide pathway TSS, as well as several internal promoters located at various regions throughout the gene cluster (Figures 4, 5 and 6). Previous studies with β-galactosidase reporter assays have been useful in demonstrating how enhancer elements within untranslated leader regions and intergenic promoters are important in driving transcription [37,43]. The strongest promoter in the assay was that identified upstream of the *jamA *TSS, but several other promoters were either equal to or greater in strength than the positive control in the assay. One of the regions predicted to contain a strong promoter (up*jamI*) is located in front of a large set of ORFs. The ORF *jamI*, encoding an enoyl-CoA hydratase/isomerase, forms a di-domain with *jamJ*, which encodes for an enoyl reductase and a large PKS [6]. In addition, the subsequent ORFs in the pathway (*jamK - M*) are separated by small intergenic regions and do not appear to contain promoters. If *jamI - M *form one contiguous transcript (~30 kb), a promoter in front of *jamI *could be needed for efficient transcription. The identification of functional promoters in several other intergenic regions suggests that they could also be used to boost transcription beyond the capacity of the initial promoter located before the TSS upstream of *jamA*.

One intriguing finding from using truncated intergenic regions in the β-galactosidase assay was the detection of strong activity immediately upstream of *jamA *(-76 - 0) and *jamI *(-67 - 0) (Figure 5). An additional promoter was predicted in a region of up*jamI *(-269 - -203) farther upstream in the 5' direction (Table 1), but this region was not active when used in truncated form (Figure 5). If these active regions upstream of *jamA *(-76 - 0) and *jamI *(-67 - 0) are able to act as internal promoters to supplement overall transcription of the jamaicamide pathway, their close proximity to *jamA *and *jamI *may compromise the ability of transcripts initiating at these positions to subsequently allow for proper translation of the JamA and JamI proteins (although transcription could take place normally downstream of each location). This could occur as a result of insufficient room for a ribosome binding site, although translation of mRNA in cyanobacteria may not require the use of Shine-Dalgarno sequences [44] and some evidence exists for translation of leaderless mRNA in bacteria [45]. It is possible that our heterologous use of these up*jamA *(-76 - 0) and up*jamI *(-67 - 0) regions in *E. coli *could lead to false positive identification of promoters in some instances. However, as previously discussed, the organization of the gene cluster supports the utility of functional promoters in both locations. The untranslated leader region of *jamA *is long enough for the presence of additional regulatory elements, and up*jamI *is a probable location for a promoter because of the long *jamI - M *transcript. Further evaluation of these two possible promoters will be necessary to determine how transcription from their locations could affect subsequent protein translation.

Of particular interest in this study was the successful isolation of proteins using "pulldown" experiments that could be involved in the regulation of jamaicamide expression. Gene clusters of marine cyanobacterial PKS/NRPS secondary metabolites identified to date lack any associated regulatory proteins that are imbedded in or in proximity to the main cluster, in contrast to antibiotic pathways in other prokaryotes such as actinobacteria [27]. This absence has led to the suggestion that secondary metabolite pathways from *L. majuscula *could be constitutively expressed [6]. By using the upstream region of *jamA *as a DNA probe, we hoped to isolate putative regulatory proteins from the soluble protein fraction of JHB. This was predicted on the hypothesis that if the jamaicamide pathway does have associated regulatory proteins, they are located elsewhere in the genome. A biotinylated DNA sequence from the jamaicamide pathway (1000 bp upstream of *jamA *to 20 bp into the *jamA *gene) was incubated with protein lysate from *L. majuscula *JHB. The probe was long enough to encompass the entire untranslated leader region of the pathway, as well as the primary promoter and an additional 123 bp upstream of the promoter -35 hexamer. Because transcription factors commonly bind at either the -35 box of the promoter itself, or within 90 bp of the -35 box [46], it is probable that the probe was long enough to capture proteins that might associate with the promoter. The probe also allowed for binding of regulatory proteins with affinity to the untranslated leader region [37,47]. Analysis of protein samples isolated from both an excised SDS-PAGE gel band and elution fractions of several repeated pulldown assays consistently identified two proteins in three separate data sets using LC-MS/MS. These proteins were partially identified using sequence data from the unfinished *L. majuscula *3L genome (unpublished), a strain from Curaçao responsible for the production of the anticancer compound curacin A [5,48]. The two proteins (5335 and 7968) displayed strongest sequence identity to hypothetical proteins found in other cyanobacteria, but could not immediately be assigned a function. BLAST searches with both proteins resulted in hits with RcaD, a protein involved in complementary chromatic adaptation (CCA) in another species of cyanobacteria [34]. Interestingly, although the level of sequence identity of the two proteins with RcaD was quite different (Table 2), both proteins (in the 3L genome) had a similar gene neighborhood to RcaD, indicating probable synteny. The *L. majuscula *3L proteins downstream of each (5336 and 7969) both had BLAST hits with RcaG, the ATPase associated with RcaD, although 7969 (49% identity) had significantly more identity than 5336 (23% identity).

Complementary chromatic adaptation has been identified in a number of freshwater [36] and marine cyanobacteria [49]. In the cyanobacterial CCA model organism *Fremyella *(= *Calothrix, Tolypothrix*), a photoreceptor circuit involving the Rca receptors and response regulators (RcaC, RcaE, RcaF, and RcaD) has been found to be responsible for pigment modifications under red and green light [35]. RcaD appears to affect several operons during the acclimation phase of CCA [34]. Although *Lyngbya majuscula *strains have not been observed to undergo CCA in culture, there are several color morphotypes known (for example, in our culture collection *L. majuscula *3L is red under 16 h light/8 h dark cycles, while *L. majuscula *JHB is dark green). In addition, a microarray analysis of cyanobacteria undergoing CCA found that over 80 genes were upregulated, including many not involved in photosynthesis [50]. Considering the widespread effects that CCA regulatory proteins play in cyanobacteria, it is plausible that secondary metabolite production is regulated by homologous proteins. Regulation by light could also be in accordance with the mechanisms previously described for the microcystin biosynthetic pathway [21,22].

To further evaluate the two possible regulatory proteins isolated in the pulldown assay, we overexpressed both proteins in *E. coli *to evaluate their respective binding affinities for the jamaicamide primary promoter region. Protein 7968 was found to bind to the proposed transcription factor binding region of the jamaicamide pathway (1000-832 bp upstream of *jamA*; Figure 9a), and this DNA binding activity was supported with serial protein titration (Figure 9b). Although we demonstrated that a control protein would not bind under the same conditions, we also found that protein 7968 was able to bind nonspecifically to several other unrelated pieces of DNA. Thus, we were unable to assign a specific sequence for 7968 binding. Attempts to cleave the GST tag from the 5335 protein were unsuccessful, and binding assays indicated that the GST+5335 fusion protein was not able to bind to the same intergenic region as 7968 (Figure 9a; Additional File 3: Figure S2). Because of its strong affinity with DNA, 7968 is the better candidate protein for providing transcriptional regulation of the jamaicamide pathway. The presence of multiple intergenic promoters in the pathway could also offer other binding locations for additional regulation.

It is difficult to predict how the binding affinity of recombinant forms of 5335 or 7968 compares quantitatively with the native proteins. Noubir et al. [34] found that native RcaD bound much more effectively to the phycocyanin 2 promoter than a recombinant version, and hypothesized that the reduced affinity may be from lack of ATPase RcaG, which facilitates binding, or from lack of phosphorylation. We attempted a dual-shift experiment with 7968 and the GST tagged 5335, but no shift differences compared to 7968 alone were observed (data not shown). It will be intriguing to determine whether 5335 and 7968 work in tandem to regulate the jamaicamide pathway, or if they require downstream neighbors (5336 or 7969) to assist in binding. Alternatively, it is possible that 7968 is the true regulator of the pathway, and 5335 was "pulled down" in the magnetic bead assay due to its sequence identity being minimally sufficient for recognition. Interestingly, protein 7968 was found to form dimers by PAGE analysis. Transcription factors often function as dimers in their association with DNA and RNAP [46], and thus, this finding also supports 7968 as the best candidate regulatory protein identified in this study.

If transcription factors are in fact regulating the expression of secondary metabolites such as jamaicamide, it is useful to consider the potential pleiotropic role of proteins such as 7968 in regulating more than one biosynthetic pathway in *L. majuscula *JHB. There are a number of similarities in the secondary metabolite gene clusters of *L. majuscula*, such as those encoding for the jamaicamides, hectochlorin (also produced by the JHB strain; [39] and curacin A [5,51]. For example, the genes *jamA *and *hctA *are both ACP synthetases and are 58% identical, which might indicate that similar regulatory proteins associate with the upstream regions of each gene. If jamaicamide and hectochlorin are both used in defense of *L. majuscula *against predation or infection, their co-regulation would enhance the defense of the strain. It is also interesting to speculate that proteins in *L. majuscula *3L homologous to jamaicamide regulatory proteins could be used to regulate production of curacin A. A comparison of the approximately 1700 bp that separate *jamA *from its upstream neighboring gene (a transposase) with the upstream region of *curA *from the curacin A pathway reveals that approximately 1550 bp of the up*jamA *region is 95% identical with the up*curA *region. Moreover, proteins 5335 and 7968 are 99.6% and 89.5% identical with their respective homologs in *L. majuscula *3L (the curacin A producer). If either of these two proteins functions as a pleiotropic regulator for natural products biosynthesis in *L. majuscula*, their use in overexpression efforts would be valuable in unlocking the full biosynthetic potential of these filamentous marine cyanobacteria.

Ultimately, quantitative co-transcription analyses of the two proteins with the rest of the jamaicamide pathway and gene knockouts will be necessary to conclusively link these proteins with jamaicamide regulation. Current efforts are evaluating transcription levels of the two proteins with both jamaicamide transcription and compound production, and the effect of variable light wavelengths on jamaicamide production in culture. Because targeted gene manipulation techniques in *L. majuscula *have not yet been developed, we are also in the process of conducting methodology experiments to disrupt or overexpress 5335 and 7968 to better understand their functions, including their roles in global regulation.

## Conclusion

Understanding the regulation of natural product pathways that encode compounds with pharmaceutical potential is important to overcoming the "supply issue" that is so prevalent in natural products research [8]. While marine cyanobacteria are recognized as prolific producers of bioactive compounds, natural product yields from field collections are low, and slow culture growth severely limits the amount of compound that can be produced in this manner. The transcriptional profile of the jamaicamide biosynthetic gene cluster presented here provides insight into the mechanisms by which these pathways are transcribed and potentially regulated. Future advances in classifying promoters and transcription factors for cyanobacterial gene clusters will be important to diverse applications in biotechnology, such as combinatorial biosynthesis and the heterologous expression of entire natural product pathways. Additionally, this information should also benefit ongoing efforts attempting to regulate the expression of cyanobacterial toxins with deleterious environmental impacts.

## Methods

### Bacterial strains, culture conditions, PCR reactions, and DNA measurements

*Lyngbya majuscula *JHB was originally collected from Hector's Bay, Jamaica [6] and was maintained in a culture facility at Scripps Institution of Oceanography. Cultures were grown in BG-11 saltwater media at 29°C under a light intensity of approximately 5 μE m^-2 ^s^-1 ^and under 16 h light/8 h dark cycles. *E. coli *TOP-10 and BL-21 (DE3) were grown in Luria-Bertani (LB) media. *E. coli *cultures were grown with ampicillin (100 μg ml^-1^), or kanamycin (50 μg ml^-1^) when necessary. PCR reactions were conducted using either PCR Master Mix (Promega) or Pfx50 proofreading Taq Polymerase (Invitrogen). DNA concentrations were measured using either Beckman-Coulter DU800 or NanoDrop 1000 (Thermo Scientific) spectrophotometers. Protein concentrations for recombinant JHB proteins were determined using the BCA assay (Pierce). Ladders for DNA (Fermentas and New England Biolabs) and protein (Bio-Rad) were used for size estimations when necessary.

### RT-PCR using *L. majuscula *RNA to search for the transcription start site (TSS) and promoter regions in the jamaicamide pathway

Cyanobacterial filaments (approximately 2 g wet weight) from a culture of the jamaicamide producing strain of *L. majuscula *JHB were harvested and subjected to RNA isolation using TRIzol reagent (Invitrogen) and procedures based on those recommended by the manufacturer with minor modifications. RNA was treated with TURBO DNAse (Ambion) for 2 h at 38°C before use in cDNA reactions. To verify that genomic DNA contamination was not present, in selected cases negative control reactions were run in parallel with cDNA reactions in which reverse transcriptase enzyme was omitted. For the primer extension experiment, first strand cDNA was synthesized from the RNA using the primer up*jamA *20-0 R (Sigma Genosys; Additional file 1: Table S1) and the Superscript III Reverse Transcriptase Protocol (Invitrogen) with minor modifications. Second strand reactions were conducted with primers ranging from 500-902 bp upstream in 50 bp increments to determine where RNA transcription upstream of *jamA *initiated. For cDNA synthesis of jamaicamide intergenic regions, first strand cDNA was generated using either random (Invitrogen and [52]; Additional file 1: Table S1) or specific jamaicamide upstream intergenic region reverse primers (Additional file 1: Table S1). Forward and reverse oligonucleotide primers for each upstream intergenic region of the cluster were used to PCR amplify regions from the first strand cDNA to create second strand cDNA. In some instances, sequencing was used to confirm the correct amplification of second strand cDNA, either by direct sequencing of PCR products or by sequencing of TOPO TA cloning vectors (Invitrogen) containing the intergenic region. All cDNA PCR products were visualized on agarose gels.

### Use of promoter prediction and β-galactosidase reporter gene assays to search for promoter activity

Each intergenic region upstream of the genes in the jamaicamide pathway was examined for conserved binding regions (in comparison to the σ^70^*E. coli *consensus promoter) using the BPROM predictor (http://www.softberry.com; Table 1). The upstream (up-) regions of genes predicted to contain a promoter (*jamA, jamB, jamC, jamD, jamG, jamI*, *jamN, and jamQ*) were amplified with specific primers (Additional file 1: Table S1) from fosmids produced previously [6]. Each upstream section was individually cloned into the pBLUE TOPO vector (Invitrogen) and transformed into TOP-10 *E. coli*. Plasmid purification (Qiagen) and sequencing (Seqxcel, Inc., La Jolla, CA) were used to confirm the sequence and direction of the inserts. Verified clones were used to measure relative promoter activity using the β-galactosidase reporter gene assay (Invitrogen), standardized against total soluble protein content as measured by BCA assay (Pierce). For this protocol, colonies from freshly streaked selective (100 μg ml^-1 ^ampicillin) plates were grown overnight in LB media (5 ml). Cultures were pelleted at 4000 RPM, and the pellets were washed once with 3 ml of chilled PBS. After an additional centrifugation step, the pellets were resuspended in 1 ml PBS. The tube contents were centrifuged at 14,000 RPM for 5 min, and subsequently resuspended in 110 μl lysis buffer (0.25 mM Tris, pH 8.0). The cells were lysed by freeze-thawing the suspensions 4 times with dry ice and a 37°C water bath, followed by another centrifugation period. The supernatant was removed from each tube for use in the β-galactosidase and BCA assays in a 96 well plate. O-nitrophenol measurements from ONPG cleavage in the assay were taken at 420 nm, while BCA readings were taken at 570 nm (Thermo-Electron Multiskan Ascent plate reader). Serial dilutions of lysis buffer from each culture were made to find an optimal range for colorimetric detection of o-nitrophenol and for a comparison of relative activity (identified as a 3-fold dilution). Relative activity calculations were made by determining the average nmol ONPG hydrolyzed min^-1 ^mg soluble protein^-1 ^value for each vector insert and dividing each value by the highest overall average value to obtain a percentage. A fragment of the pBAD TOPO vector (Invitrogen) containing the arabinose promoter and vector ribosome binding site (upstream of the TOPO cloning site) from *E. coli *was found to consistently produce β-galactosidase in the pBLUE TOPO vector in preliminary experiments, and was used as a positive control. Because the arabinose operator was not included in the positive control, the addition of arabinose was not required to produce β-galactosidase. A 49 bp segment of the jamaicamide *jamG *gene was used as a negative control. [Note: the pBLUE vector contains a cryptic promoter that is reported to possibly limit the efficacy of assaying other promoter fragments in a prokaryotic host (Invitrogen). However, a series of preliminary assays indicated significant and repeatable differences in promoter activity between possible promoter regions, and baseline activity in the negative control was sufficiently low as to not conflict with the assay results. The BPROM prediction software was used to verify that the vector constructs did not introduce any artificial promoters]. Those regions found to have promoter activity were assayed again with additional dilution (10 fold) to quantify promoter strength, expressed as specific activity (nmol ONPG hydrolyzed min^-1 ^mg soluble protein^-1^).

### Isolation of possible transcription factors from a pulldown assay

Protein pulldown experiments were based on methods similar to [53]. A DNA probe that extended from 1000 bp upstream of *jamA *to 20 bp into the *jamA *gene was amplified by PCR from the jamaicamide fosmid described above using the primers up*jamA *1000 biotin (biotinylated at the 5' end; Invitrogen) and up*jamA *20 - 0 R (Additional file 1: Table S1). The PCR product was purified (MinElute PCR Purification Kit, Qiagen) and 10 pmol of the biotinylated DNA were incubated with 1 mg of magnetic M-270 streptavidin Dynabeads (Invitrogen), according to the manufacturer's instructions. *L. majuscula *JHB tissue was obtained from pan cultures that had been growing for 1-2 months. Approximately 2-3 ml of culture was measured by displacement in sterile, chilled binding buffer [10 mM Tris-HCl (pH 7.5), 1 mM EDTA, 1 mM DTT, 150 mM NaCl, and 5% (w/v) glycerol]. The binding buffer was also treated with a broad range protease inhibitor (Complete, EDTA free; Roche). The tissue was sonicated and kept on ice using a probe sonicator with six 10-s pulses, and insoluble material was pelleted at 13,200 RPM for 10 minutes. The soluble protein fraction (750 μl) was added to each mg of DNA coated beads. One μg of Poly DI-DC was also added to inhibit non-specific binding of protein to the DNA. Magnetic beads that were not treated with biotinylated DNA were incubated with JHB soluble protein as a negative control. The beads and soluble protein were incubated for 1 h using an end-over-end rotator at 4°C. The beads were subsequently washed twice using 200 μl of binding buffer containing 100 μl sheared salmon sperm DNA (Invitrogen; 5 mg ml^-1^), three times with binding buffer, and eluted with 50 μl of binding buffer containing 1.0 M NaCl. Wash fractions and elutions were flash frozen in liquid N_2 _and concentrated on a freeze dryer. After concentration, aliquots of each were mixed with protein sample buffer, denatured for 3 minutes at 95-100°C, and analyzed by SDS-PAGE. The gels were stained with either silver (Silverquest Kit, Invitrogen) or colloidal Coomassie brilliant blue G-250.

### Identification of DNA binding proteins

Once gel bands were visible in the elution fraction from the binding assay, the assay was repeated on a larger scale using additional replicates of the procedure described above to isolate sufficient protein for mass spectrometry (visible by colloidal Coomassie staining). Both gel bands (excised using a scalpel) and whole elution fractions were submitted to The Scripps Research Institute (La Jolla, CA) Center for Mass Spectrometry for nano-LC MS/MS analysis. Raw spectrum data (mzdata format) was obtained and analyzed at UCSD by a DOS common-line version of InsPecT 20070712 [31].

InsPecT search parameters for the mzdata files were the following: (i) *Lyngbya majuscula *3L common database (unpublished data), common contaminants database, reverse or "phony" database, and NCBI nr database; (ii) parent ion Δm = 1.5 Da; (iii) b and y-ion Δm = 0.5 Da. Top protein identifications were verified by using two different database searches: (i) *Lyngbya majuscula *3L genome alone; (ii) NCBI nr with *L. majuscula *3L genome inserted. The mass spectral identifications of 5335 and 7968 were further verified by manual annotation of the N-terminal and C-terminal peptides, as well as the most abundant peptide identified.

### Characterization of putative transcription factors from a pulldown assay

Protein sequences detected using InsPecT were compared with raw nucleotide sequences from the *L. majuscula *3L genome to identify their corresponding ORFs. Forward and reverse primers (5335 F &R, 7968 F &R, Additional file 1: Table S1) were designed from each sequence and used to amplify the corresponding genes from *L. majuscula *JHB. The blunt PCR products were cloned (Z-Blunt TOPO vector, Invitrogen) and transformed into *E. coli *for sequencing to compare the gene sequences from JHB with those of 3L. Additional gene boundary primers (5335 FB, 5335 RB; 7968 FB, 7968 RB; Additional file 1: Table S1) were used to amplify the JHB genes with priming sites 25 bp upstream and downstream in order to verify the sequences covered by 5335 and 7968 forward and reverse primers and avoid inclusion of sequences from *L. majuscula *3L. Bioinformatic analyses of each gene sequence were conducted using BLAST programs available through the National Center for Biotechnology Information (NCBI; http://blast.ncbi.nlm.nih.gov/).

### Recombinant expression of identified proteins

Genes corresponding to identified proteins in the JHB protein pulldown assay were amplified from JHB genomic DNA using the primers 5335 Nco1F and 5335 Not1R or 7968 Nde1F and 7968 Xho1R (Additional file 1: Table S1). Start codons in both gene sequences were changed from either leucine (5335) or valine (7968) to methionine for improvements in *E. coli *overexpression efforts. PCR products from both reactions were purified and digested with their corresponding restriction enzymes (New England Biolabs). Gene 5335 was ligated into the pGS21a vector (Genscript), which contains both an N-terminal 6× His tag and GST tag. The 5335 construct was verified via transformation, plasmid isolation from TOP-10 *E. coli*, and sequencing. The vector containing the gene sequence was then transformed into BL-21 (DE3) *E. coli*. Four liters of *E. coli *harboring the 5335 pGS21a vector were grown (using starter cultures) for 4 h at 37°C to an OD_600 _between 0.6 and 1.0, induced with 0.7 mM IPTG, and then grown at 18°C overnight. Cultures were centrifuged at 4500 RPM for 15 min at 4°C, and the ensuing pellets from each liter of culture were resuspended in 5 ml protein lysis buffer (20 mM Tris, pH 8.0, 500 mM NaCl, and 20 mM imidazole) and lysed on ice using sonication (6-7 10-s pulses). Resuspended lysate was centrifuged, and supernatant containing soluble protein was incubated on an end-over-end rotator at 4°C with Nickel-Superflow resin (Qiagen) for 2 h. Following incubation, the recombinant GST+5335  fusion protein was purified using polypropylene columns (Qiagen). The nickel slurry from the incubation was washed twice with protein wash buffer (20 mM Tris, pH 8.0, 500 mM NaCl, and 50 mM imidazole), and protein was eluted with 5 × 1 mL aliquots of protein elution buffer (20 mM Tris, pH 8.0, 500 mM NaCl, and 750 mM imidazole). Purified protein was dialyzed against binding buffer (the same buffer used for the pulldown assay, but not containing DTT) overnight using a 50 kDa MWCO dialysis membrane (Spectrum Labs, Rancho Dominguez, CA). To express 7968, the corresponding gene sequence was ligated into the N-terminal His tag containing pET28b vector (Novagen), and grown and purified similarly to 5335 (although the 7968 wash buffer contained 40 mM imidazole). Spectra/Por Float-A Lyzer G2 dialysis membranes (20 kDa MWCO; Spectrum Labs) were used to dialyze protein 7968. Concentrations of each protein for use in assays were determined using the BCA assay (Pierce).

### Electromobility shift assays (EMSAs)

Gel shift EMSAs were performed to verify binding of 5335 and 7968 to jamaicamide promoter regions. The region upstream of the jamaicamide TSS (1000 - 832 bp upstream of *jamA*) was amplified from a jamaicamide fosmid using Pfx50 Taq Polymerase (Invitrogen). Each PCR product was purified (MinElute kit, Qiagen) before being used in the assay. For the comparative binding assay (Figure 9a), the N-terminal His tag was cleaved from protein 7968 using the Thrombin Cleavage Capture Kit (Novagen). The cleaved 6× His tag was subsequently removed by concentrating the protein sample over a Microcon 10,000 MWCO column (Millipore). SDS-PAGE gels and western blotting were conducted to confirm the success of the cleavage reaction. Serial titration of the 7968 protein to verify DNA binding was performed using the recombinant version of the protein without His tag cleavage. GST+5335 elutions were also concentrated over Microcon 10,000 MWCO columns prior to use in shift assays. For a negative control, the purified recombinant protein HctE_IVA _from the hectochlorin pathway (purification described in [39]) was used. The concentrations of HctE_IVA _protein used in the EMSA experiments were measured using a Bradford assay, and the purified HctE_IVA _included a 6× N-terminal His tag from its expression vector (pET28b; Novagen). Each gel shift assay reaction was performed with the indicated quantities of DNA and purified protein (Figure 9) in EMSA binding buffer adapted from the DIG Gel Shift Kit, 2^nd ^generation protocol (Roche) [20 mM HEPES, pH 7.6, 1 mM EDTA, 10 mM (NH_4_)_2_SO_4_, 1 mM DTT, Tween 20, 0.2% (w/v), 30 mM KCl] and water (total volume 20 μl) for 30 min at room temperature. Following the incubation period, 5 μl of native loading dye containing bromophenol blue was added to each reaction, and the reaction contents were immediately transferred to a 10% native PAGE gel. The gels were electrophoresed at 85 V for ~3.0 h in 0.5× TBE buffer (44.5 mM Tris, 44.5 mM boric acid, 1 mM EDTA), followed by staining for at least 10 min in SYBR Gold Nucleic Acid Gel Stain (Molecular Probes/Invitrogen) and visualization on a UV transilluminator.

## Sequence information

DNA and amino acid sequences of the proteins identified in this study have been deposited in Genbank under the accession numbers GQ860962 and GQ860963.

## Authors' contributions

ACJ, LG, and WHG conceived of the study and designed experiments, ACJ performed experiments and drafted the manuscript, and DG and PCD performed protein mass spectrometry analyses. All authors contributed to, read, and approved the final manuscript.

## Supplementary Material

Additional file 1 **Table S1: Primers used in this study**. This Excel file (.xls) is a complete list of all primers used for RT-PCR experiments, β-galactosidase reporter assays, and protein identification, recombinant expression, and EMSA experiments. Click here for file

Additional file 2 **Figure S1: Sequence alignment with *Lyngbya majuscula *JHB protein 7968 and 5 proteins with highest identity matches from NCBI BLAST analyses**. This TIFF file (.tiff) shows an alignment of these 6 protein sequences performed in ClustalX2. Click here for file

Additional file 3 **Figure S2: EMSA with DNA region -1000 - -832 bp upstream of *jamA *and protein GST+5335**. This TIFF file (.tiff) shows, from left to right: 270 fmol DNA only, 8.4 pmol, 16.4 pmol, 33.5 pmol, and 67.0 pmol of GST+5335 combined with 270 fmol DNA. Click here for file
