# Supplementary figures and images for: Transcriptional analysis of the jamaicamide gene cluster from the marine cyanobacterium Lyngbya majuscula and identification of possible regulatory proteins
Source: BMC Microbiol. 2009 Dec 1;9:247. doi: 10.1186/1471-2180-9-247 (PMC2799420; doi:10.1186/1471-2180-9-247)

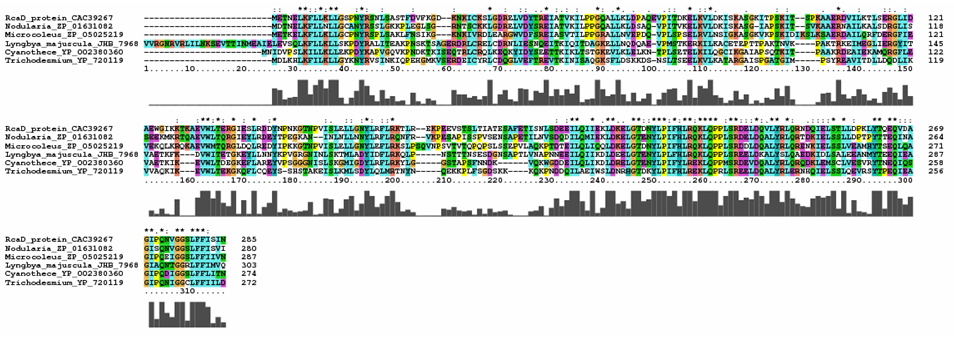

Supplement: Additional file 2 — Figure S1: Sequence alignment with Lyngbya majuscula JHB protein 7968 and 5 proteins with highest identity matches from NCBI BLAST analyses. This TIFF file (.tiff) shows an alignment of these 6 protein sequences performed in ClustalX2. [file 1471-2180-9-247-S2.TIFF]

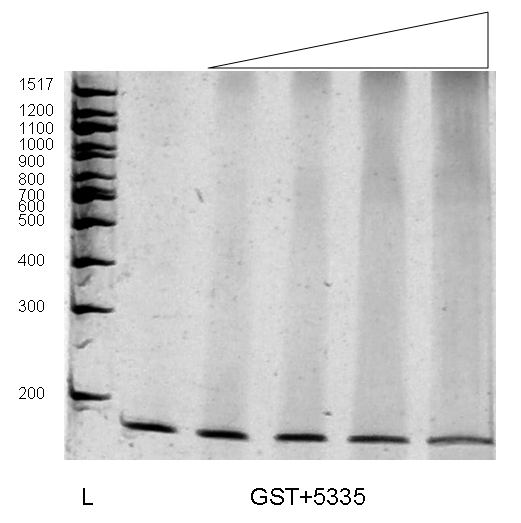

Supplement: Additional file 3 — Figure S2: EMSA with DNA region -1000 - -832 bp upstream of jamA and protein GST+5335. This TIFF file (.tiff) shows, from left to right: 270 fmol DNA only, 8.4 pmol, 16.4 pmol, 33.5 pmol, and 67.0 pmol of GST+5335 combined with 270 fmol DNA. [file 1471-2180-9-247-S3.TIFF]
